# High-Accuracy Characterization of a Single Thin Film on a Substrate from One Transmittance Spectrum by an Advanced Envelope Method Addressing Voids, Tail Electron Transitions, and Deep-Level Electron Transitions in a-Si Films

**DOI:** 10.3390/nano16090522

**Published:** 2026-04-26

**Authors:** Dorian Minkov, George Angelov, Dimitar Nikolov, Rostislav Rusev, Manuel Ballester, Susana Fernandez, Emilio Marquez

**Affiliations:** 1 Scientific Research Section (NIS), Technical University, 1000 Sofia, Bulgaria; 2 Department of Microelectronics, Faculty of Electronics Engineering and Technologies, Technical University, 1000 Sofia, Bulgaria; angelov@ecad.tu-sofia.bg (G.A.); dnikolov@elsys-bg.org (D.N.); 3 Department of Technology and Management of Communication Systems, Faculty of Telecommunications, Technical University, 1000 Sofia, Bulgaria; rusev@ecad.tu-sofia.bg; 4 Computer Science Department, Northwestern University, Evanston, IL 60208, USA; manuelballestermatito2021@u.northwestern.edu; 5 Photovoltaic Solar Energy Unit, Centre for Energy, Environmental and Technological Research (CIEMAT), Avenida Complutense 40, 28040 Madrid, Spain; susanamaria.fernandez@ciemat.es; 6 Faculty of Science, Department of Condensed-Matter Physics, University of Cadiz, Puerto Real, 11510 Cadiz, Spain; emilio.marquez@uca.es

**Keywords:** thin film characterization, optical properties, UV-Vis-NIR transmittance spectrum, internal nanoscale surface structure, superior denoising, advanced envelope method, volume fraction of voids, a-Si films, Urbach tail, dangling bonds

## Abstract

In most amorphous materials, the concentration of Urbach tail states is larger than the concentration of dangling bond states. However, absorption accounting for the Urbach tail while disregarding the dangling bonds is commonly used or derived by spectroscopic characterizations of amorphous films from a single spectrum, mostly due to the insufficient accuracy of such characterizations. This paper proposes an advanced envelope method (AEM) for transmittance spectrum *T*(*λ*), aiming to resolve this problem. The novelties in AEM are: improved preprocessing of *T*(*λ*), extending the envelopes deeper into the region of strong absorption (RSA), enhanced determination of the refractive index *n*(*λ*) in the region of weak absorption, optimization of both *n*(*λ*) and the extinction coefficient *k*(*λ*) in RSA, as well as analysis of the types of electron transitions and calculation of their energy gaps. Three single magnetron sputtered a-Si films deposited on glass substrates are characterized by AEM, and three other relevant methods that disregard deep-levels. The best accuracy is achieved when these films are characterized by AEM. It is demonstrated that the absorption coefficient *α*(*λ*) of each of these films distinguishes electron transitions via dangling bond states from those via tails states, and the DOS corresponds to the Mott–Davis model of amorphous materials.

## 1. Introduction

The most practical applications of materials in optics, optoelectronics and electro-optics use light from the UV-Vis-NIR spectral region [1,2,3]. Moreover, the bandgap *E_g_* of the vast majority of semiconductor and dielectric materials is within this spectral region [4,5,6]. This means that thin films from these materials can be both semi-transparent and opaque when illuminated by such light. In addition, the characteristics of thin films often depend on the technology for their preparation [7,8,9]. These factors determine the extensive use of characterization of a single thin film, deposited on a substrate, by utilizing either spectroscopic ellipsometry or spectrophotometry [10,11]. In this type of characterization, the thickness *d_s_* of the substrate (regarded as constant), the refractive index *n_s_*(*λ*), and the extinction coefficient *k_s_*(*λ*) are known, where *λ* is the wavelength. The thickness *d* of the film can vary over the light spot, so that *d* ⊂ [$\bar{d}$ − ∆d, $\bar{d}$ + ∆d] where $\bar{d}$ is the average film thickness over the light spot and ∆d > 0 is the non-uniformity of the film. The dielectric properties of the film are determined by its refractive index *n*(*λ*) and extinction coefficient *k*(*λ*) ≥ 0.

In this paper we study magnetron sputtered unhydrogenated a-Si semiconductor films. It is expected that such films contain tail states with concentrations in the order of 10^3^ times smaller than those of the Si atoms, as well as deep level states (mostly dangling bond states [12]) with concentrations significantly smaller than those of the tail states [13]. The main methods for analysis of dangling bonds are: electron spin resonance, the constant photocurrent method, photothermal deflection spectroscopy, and the defect pool model [14]. Thin a-Si films have been parametrized by using spectroscopic ellipsometry [15,16,17,18]. However, there are no published parametrizations of a-Si films by spectroscopic ellipsometry in the UV-Vis-NIR region, nor by spectrophotometry in the same region, accounting for both the tail states and dangling bond states. The main reasons for this are likely the relatively low concentrations especially of the dangling bonds in a-Si, noise in the measured spectra, and the computational difficulties associated with these. However, ignoring the deep-level states results in incorrect analysis of the subgap electron transitions (for photon energy *E* (eV) = 1239.8/*λ* (nm) < *E_g_*) and the subgap behavior of the absorption coefficient α(*λ*) = 4π*k*(*λ*)/*λ*.

The normal incidence transmittance spectrum *T*(*λ*) of the sample, consisting of a thin film on a substrate, for light incident onto the film, is commonly measured by a UV-Vis-NIR spectrophotometer [9,19]. The substrate is commonly selected to be thicker than the coherence length for the film, which prohibits substrate interference features in *T*(*λ*) [20,21]. In addition, the average thickness of thin film is usually smaller than the coherence length for the film, thus allowing the occurrence of thin film interference features in *T*(*λ*) [22,23]. In general, the unknowns in characterizations of single thin films are: $\bar{d}$, ∆d, *n*(*λ*), and *k*(*λ*) [24,25].

However, spectrophotometric measurements of such *T*(*λ*) contain noise which can decrease the accuracy of film characterization, as the predominant types of noise are: outliers in *T*(*λ*), general noise, and bandpass noise [26,27]. Therefore, preprocessing of the measured spectrum could be performed to eliminate the discussed-above three types of noise from *T*(*λ*), thus obtaining a preprocessed transmittance spectrum *T*_p_(*λ*) to be utilized thereafter.

In this paper we only study not-too-thin a-Si semiconductor films with $\bar{d}$ > *λ*/(2*n*), since *T*(*λ*) of such films has at least one interference extremum at *λ* > *λ*_g_ (nm) ≈ 1239.8/*E_g_* (eV) [28]. Provided that *T*_p_(*λ*) contains several interference maxima and minima, a higher envelope *T*_+_(*λ*) ≥ *T*_p_(*λ*) and a lower envelope *T*_−_(*λ*) ≤ *T*_p_(*λ*) can be drawn around *T*_p_(*λ*), after which envelope methods (EMs) can be used for characterization of the respective film. Well-established explicit formulae about *T*_p_(*λ*), *T*_+_(*λ*), and *T*_−_(*λ*) for a non-uniform thin film on a non-transparent substrate are presented in [29], based on derivation of *T*_p_(*λ*) from [30].

Since all EMs developed for *T*_p_(*λ*) are based on using both its higher and lower envelopes, accurate film characterization by such EM requires the accurate drawing of *T*_+_(*λ* > *λ*_c_) and *T*_−_(*λ* > *λ*_c_), where *T*_p_(*λ*_c_) is an adjustable convergence point of the envelopes, so that *T*_+_(*λ* < *λ*_c_) ≡ *T*_−_(*λ* < *λ*_c_). With regard to this, the interpolation iterative algorithm of McClain et al. [31] has been used for drawing envelopes of *T*_p_(*λ*) by several authors [32,33]. Yet, the algorithm from [31] does not include boundary points of the envelopes at the longest wavelength max(*λ*) of *T*_p_(*λ*); consequently, explicit expressions for *T*_+_(max(*λ*)) and *T*_−_(max(*λ*)) were proposed for drawing envelopes of *T*_p_(*λ*) in the case of transparent substrate [34]. However, the expression for *T*_p_(*λ*) is proportional to the substrate absorbance *x*_s_(*λ*) = 4π*k*_s_/*λ*, which means that the envelopes *T*_+_(*λ*) and *T*_−_(*λ*) are distorted when the substrate is non-transparent [35]. Therefore, an iterative algorithm utilizing a double transformation of *T*_p_(*λ*) was proposed, by Minkov et al. [36], for designing *T*_+_(*λ*) and *T*_−_(*λ*) in the case of non-transparent substrate. In [36], smooth envelopes are drawn around *T*_p_(*λ*)/*x*_s_(*λ*), thus excluding the substrate absorption, followed by calculating *T*_+_(*λ*) and *T*_−_(*λ*) via multiplying these smooth envelopes by *x*_s_(*λ*). Notably, the algorithm from [36] was designed for drawing accurate envelopes of *T*_p_(*λ*) in the region of weaker absorption in the film (RWA), which is defined approximately as *λ > λ*_g_, whereas the algorithm was used only for *T*_p_(*λ*) > 0.03. In addition, last year a paper was published by Ballester et al. [37], demonstrating the drawing of high-accuracy envelopes with a convergence point *T*_p_(*λ*_c_) > 0.03, for uniform film on quasi-transparent substrate, by means of a global optimization algorithm. Importantly, though, no algorithm has been reported for drawing *T*_+_(*λ*) and *T*_−_(*λ*), with a convergence point *T*_p_(*λ*_c_) << 0.03, for a non-uniform film on a non-transparent substrate. However, attaining such low *T*_p_(*λ*_c_) would allow more accurate film characterization in the region of stronger absorption in the film (RSA), defined approximately as *λ* < *λ*_g_.

The wavelengths of the tangency points between *T*_p_(*λ*) and its envelopes *T*_+_(*λ*) and *T*_−_(*λ*) are designated here as *λ*_t_, *l* = 1, 2, … *l*_M_ is the tangency wavelength number counted from the longest wavelength max(*λ*), and *m_l_*(*λ*_t_) > 0 is the order of the interference fringe. Regarding the main features of EMs, the pioneering EM of Swanepoel was developed for uniform film and transparent substrate [38]. Soon thereafter, Swanepoel published an EM for non-uniform film on a transparent substrate [39]. The first EM for the general case of a non-uniform film on a non-transparent substrate was proposed by Marquez et al. [40]. However, the EM algorithms from [38,39,40] assume the presence of a wide region of weak film absorption in *T*(*λ*), as well as relying on subjective selection of both ∆d and the boundaries of the interval of consecutive tangency wavelengths *λ*_t_ to be used. These problems were resolved by the optimizing envelope method, abbreviated as OEM and developed by Minkov et al. [41,42]. The OEM computes $\bar{d}$, ∆d, *m*_1_ = *m_l_*_=1_(*λ*_t_), as well as the tangency wavelength numbers *l*_1_ and *l*_2_ ≥ *l*_1_ + 4 corresponding to the right and left boundaries of the interval *λ* = [*λ*_t_(*l*_2_),*λ*_t_(*l*_1_)] including only the employed consecutive *λ*_t_, by minimization of the error metric:

(1) $$RE\left(\bar{d}\left(i,l_{1},l_{2}\right)\right)\left(\%\right)=\frac{l_{2}-l_{1}+2}{\left(l_{2}-l_{1}+1\right)}\sqrt{\frac{\sum_{l=l_{1}}^{l_{2}}{\left[{\bar{d}}_{e}\left(i,l_{1},l_{2}\right)-d_{a}\left(i,l\right)\right]}^{2}}{l_{2}-l_{1}+1}}\frac{100}{d_{a}\left(i,l\right)}\left(\%\right)\geq 0,$$

where *d_a_*(*i*,*l*) is an approximated value of the average film thickness for an iteration number *i* and *λ* = *λ*_t_(*l*), while ${\bar{d}}_{e}$ is an average value of *d_a_*(*i*,*l*) over the interval *l* = [*l*_1_,*l*_2_]. In addition, the minimum value of the left side of Equation (1) is designated as RE($\bar{d}$).

After $\bar{d}$ and *m*_1_ are computed by one of the above-mentioned EMs, approximated values of the refractive index *n*_a_(*λ*_t_(*l*)) = *n*(*λ*_t_(*l*)) are calculated from Equation (1) for all tangency wavelengths *λ*_t_(*l* = [1, 2, … *l*_M_]). On the other hand, it has been stated by Wemple and DiDomenico [43,44] that the refractive indices of covalent, ionic, glassy, and amorphous semiconductor materials obey the following dispersion model (DM):

(2) $$n\left(E\right)=\sqrt{1+\frac{E_{0}E_{d}}{{E_{0}}^{2}-E^{2}}},$$

corresponding to an undamped single oscillator, where *E*_0_ is the single oscillator energy, and *E_d_* is the dispersion energy which is proportional to the intensity of its underlying electron transition. With regard to this, *E*_0_ and *E_d_* are commonly determined, for thin films from such materials, by a linear regression from a Wemple–DiDomenico plot (WD plot) depicting (*n*^2^ − 1)^−1^ as a function of *E*^2^ [44]. Many EM-based studies use *n*(*λ*) of thin films calculated by replacing these *E*_0_ and *E_d_* in Equation (2) and its extrapolation over the entire range [min(*λ*),max(*λ*)] of the measured spectrum *T*(*λ*) [45,46,47]. However, such an approach is inaccurate in a RSA, since damped oscillations prevail due to intergap light absorption creating electron–hole pairs. Moreover, despite the statement of Wemple and DiDomenico about the widespread validity of DM from Equation (2), representing an undamped single oscillator, it might be possible that more than one undamped oscillator determines the behavior of *T*_p_(*λ*) in the RWA. Furthermore, after *n*(*λ*) is determined, most accurate smooth dependence *k*(*λ*) can be computed from *T*_α_(*λ*) = [*T*_+_(*λ*) *T*_−_(*λ*)]^1/2^, as shown in [42].

One technique for the computation of both *n*(*λ*) and *k*(*λ*) in RSA has been formulated for the case of transparent substrate by Swanepoel [39], so that *n*(*λ*) is represented by a first-order Cauchy equation *n*(*λ*) = *C*_1_ + *C*_2_/*λ*^2^ and *k*(*λ*) is a function of both *T*_+_(*λ*) and *T*_−_(*λ*). Nevertheless, we are not aware about this technique being used in any published research, most likely due to an inability to draw envelopes with a low convergence point *T*(*λ*_c_) << 0.03.

In the framework of EMs, the bandgap *E_g_* is usually determined from the Tauc equation [48]:

(3) $$\begin{array}{l} {\left(\alpha E\right)}^{1/q}=Q\left(E-E_{g}\right),\\ \mathrm{as}\;q=1/2\;\mathrm{for}\mathrm{direct}\;\mathrm{allowed}\;\mathrm{electron}\;\mathrm{transitions},\;q=3/2\;\mathrm{for}\;\mathrm{direct}\;\mathrm{forbidden}\;\mathrm{electron}\;\mathrm{transitions},\\ q=2\;\mathrm{for}\;\mathrm{indirect}\;\mathrm{allowed}\;\mathrm{electron}\;\mathrm{transitions},\;q=3\;\mathrm{for}\;\mathrm{indirect}\;\mathrm{forbidden}\;\mathrm{electron}\;\mathrm{transitions}, \end{array}$$

where *Q* is a proportionality constant. Moreover, a drawing depicting a dependence of the Tauc function (*αE*)^1/*q*^ vs *E* is known as a Tauc plot [49]. With regard to the above, the interval *IE* of photon energy over which the Tauc function is linear, for a particular value of *q*, corresponds to dominance of electron transitions with this value of *q*. Nevertheless, using the Tauc plot on its own is associated with the following drawbacks: *q* is assumed to have a value only amongst those shown in Equation (3), and *E_g_* can be calculated inaccurately because the interval *IE* is not well defined. These problems can be alleviated based on a result, from [50], demonstrating that *q* equals the slope of either of the curves log_10_(*αE*) vs. *E* or −d[log_10_(*αE*)]/d*E* vs. *E*, for any type of electron transition in UV-Vis spectroscopy. In addition, this result makes it possible to determine energy gaps for transitions of electrons other than from the valence band to the conduction band, as well as to identify mixed types of electron transitions with values of *q* not included in Equation (3). However, the above two curves can have large slopes over small intervals *IE*, which can lead to errors in the calculation of the interval *IE* and its respective energy gap, especially for large values of *q*.

On the other hand, amorphous materials are commonly considered to exhibit the Urbach tail in the RWA, which can be expressed as:

(4) $$\log_{10}\left(\alpha \left(E\right)\right)=\log\left(\alpha \left(E\to 0\right)\right)+\frac{E}{\ln\left(10\right)E_{U}}\;\mathrm{for}E<E_{g},$$

where *E*_U_(eV) is the Urbach energy quantifying the energetic disorder in the band edges. Therefore, it is reasonable to characterize thin amorphous films by dispersion models (DMs) including the Urbach tail, while using the previously mentioned formula about *T* (*λ*) from [30]. One such DM is the Tauc–Lorentz–Urbach model (TLU) of Foldina, employing an Urbach tail equivalent to the one described by Equation (4) [51]. Another possibility is the universal dispersion model (UDM) of Franta, where the Urbach tail occurs only for *λ* < 2*λ*_g_ due to assuming Fermi level *E*_F_ ≈ *E_g_*/2 and electron transitions from localized valence states to the conduction band as well as from the valence band to unoccupied localized states [52].

After $\bar{d}$, ∆d, *n*(*λ*) and *k*(*λ*) are determined, their replacement in the already discussed formula about the transmittance spectrum, from [30], provides a reconstructed spectrum *T*_r_(*λ*). Therefore, the following figure of merit,

(5) $$FOM\left\{\left[\lambda (j_{2}),\lambda (j_{1})\right]\right\}=1000\times \sqrt{\frac{\sum_{j=j_{1}}^{j_{2}}{\left[T_{r}\left(\lambda (j)\right)-T_{p}\left(\lambda (j)\right)\right]}^{2}}{\left(j_{2}-j_{1}+1\right)}}\geq 0$$

can be utilized as a measure of the error of the reconstructed spectrum *T*_r_(*λ*) over the interval *λ* ⊂ [*λ*(*j*_1_),*λ*(*j*_2_)], where *j* is the successive number of *λ* counted from max(*λ*). In other words, smaller *FOM*s correspond to more accurate characterizations of a thin film from its *T*(*λ*).

Furthermore, according to Bruggeman’s effective medium approximation (B-EMA), the dielectric function *ε* of a medium representing a mixture of N_y_ different media, with dielectric functions *ε*_y_, obeys the equation:

(6) $$\sum_{y=1}^{N_{y}}f_{y}\left(\frac{\varepsilon_{y}-\varepsilon }{\varepsilon_{y}+2\varepsilon }\right)=0,$$

where *f*_y_ are the volume fractions of the constituent media, whereby $\sum_{y=1}^{N_{y}}f_{y}=1$ [53]. Regarding magnetron sputtered a-Si films, they usually contain gas-filled microvoids and nanovoids, according to [54]. Taking into account that $\varepsilon \left(E\to 0\right)≅{\left[n\left(E\to 0\right)\right]}^{2}$ and $\varepsilon_{\mathrm{gas}}\left(E\to 0\right)≅1$, reworking Equation (6) for such a-Si film and *E* → 0 leads to the following expression:

(7) $$f_{\mathrm{void}}\;≃\;\frac{\left(1+\;2{n_{0}}^{2}\right)\left({n_{y0}}^{2}-{n_{0}}^{2}\right)}{3{n_{0}}^{2}\left({n_{y0}}^{2}-1\right)}\times 100(\%),$$

where *f*_void_ is the volume fraction of microvoids and nanovoids in the film, *n*_0_ = *n*(*E* → 0) is the static refractive index of the film containing voids, and *n*_y0_ = *n*_y_(*E* → 0) is the static refractive index of pure a-Si without voids [54]. Equation (7) can be used for determination of *f*_void_ of a-Si films because *n*_0_ can be obtained by replacing *E*_0_ and *E_d_* in Equation (2), while *n*_y0_ = 3.697 is derived from data about pure a-Si without voids from [55].

Based on the above, a-Si films are typically nanostructured. However, further to comments from the second paragraph, there are no published parametrizations of such films by spectroscopic ellipsometry in the UV-Vis-NIR region, nor by spectrophotometry in the same region, that simultaneously analyze their voids, tail states, and dangling bond states.

The aim of this study is to develop an advanced envelope method (AEM) to increase the accuracy of characterization of thin films from their UV-Vis-NIR *T*(*λ*), and to use it to address the above-mentioned research gap. The proposed AEM includes the following novelties: improved preprocessing of *T*(*λ*), drawing envelopes deeper into RSA, using two lines in the WD plot for the computation of *n*(*λ*) in RWA, and employing both a Cauchy equation and a new formula for determination of *n*(*λ*) and *k*(*λ*) in RSA. The performances of OEM, AEM, TLU and UDM are compared for three magnetron sputtered a-Si films, which were characterized in [54] by the EM of Swanepoel for uniform film on a transparent substrate [38]. It is demonstrated that the AEM provides more accurate characterization of these films compared to the OEM, TLU, and UDM. The superior accuracy of the AEM made it possible to simultaneously analyze voids, tail-state electron transitions, and deep-level electron transitions in a-Si films, unlike all other spectrophotometric methods for the UV-Vis-NIR region.

## 2. Materials and Methods

The studied a-Si films were prepared by using MVSystem rf magnetron sputtering deposition system. The sputtering target was p-type Si from Kurt J. Lesker Company (Jefferson Hills, PA, USA) with a size of 3.00-in. diameter × 0.250-in. thickness, a purity of 99.999%, a bulk electrical resistivity of 0.005–0.020 Ω cm, and a theoretical mass density of 2.32 g/cm^3^. Prior to the magnetron-sputtering deposition process, the glass substrates were ultrasonically cleaned. The distance between the substrate and the target was set to a convenient 6.1 cm, in order to be able to grow reasonably uniform films. Ar with a purity higher than 99.9999% was used as working gas in the sputtering process. All the depositions were performed at room temperature, and the value of the rf power applied was 525 W. The resulting rf-power density applied to the Si sputtering target was 2.9 W/cm^2^ [54]. Notably, obtaining a-Si films via magnetron sputtering is important because it provides higher film density and adhesion, as well as a low-temperature alternative, compared to those of thermal evaporation and Plasma-Enhanced Chemical Vapor Deposition (PECVD) [56,57].

## 3. Results

### 3.1. Theory and Algorithm of the Proposed Advanced Envelope Method (AEM)

In this work, the spectral dependencies of the refractive index *n*_s_(*λ*) of the substrate and its extinction coefficient *k*_s_(*λ*) are computed from *T*_s_(*λ*) and *R*_s_(*λ*), as in [39], which makes it possible to include substrate absorption. This introduces more accurate optical characteristics of the substrate, compared to the study of these films in [54] where *n*_s_(*λ*) was computed only from *T*_s_(λ), ignoring *k*_s_(*λ*), thus assuming transparency of the substrate.

**Figure A1:**
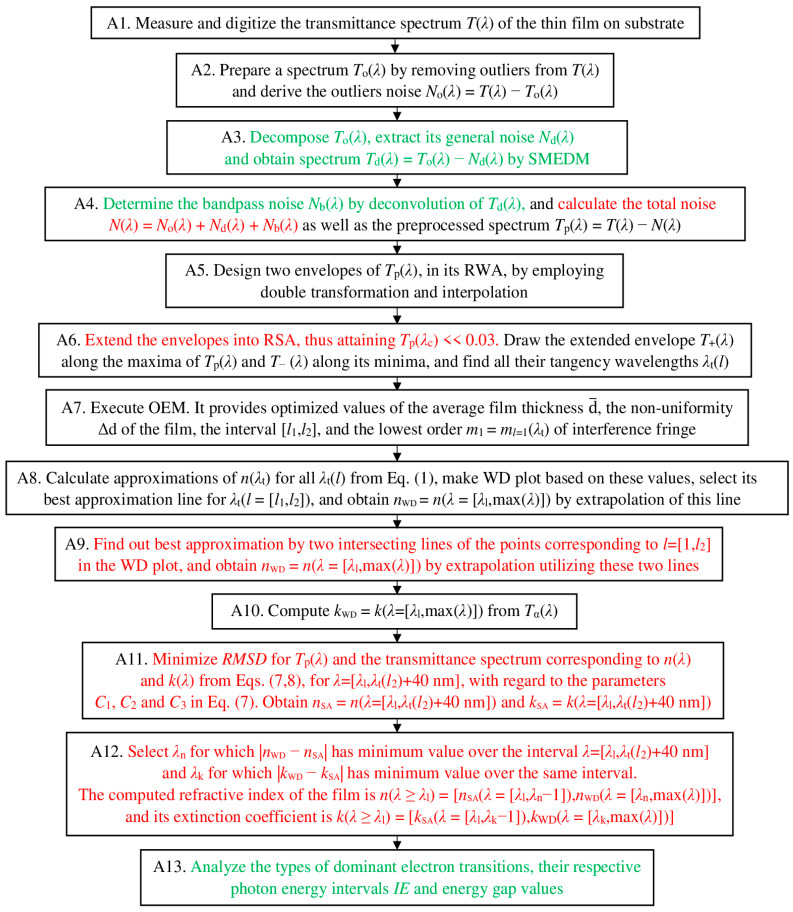
Algorithm of AEM for characterization of thin films from their *T*(*λ*). The text in green represents elements which have not been published in studies including thin film characterization, while the text in red highlights novelties of this algorithm. Steps A1 and A2 are executed as in [60], step A5— as in [36], steps A7 and A8—as in [42], and a technique from [50] is used at step A13.

In addition, AEM and OEM are used here for wavelengths *λ* > *λ*_p_, where *T*_p_(*λ*_p_) ≈ 2|*N* (*λ*_p_)| and *N*(*λ*) is the total noise, because it is not plausible to draw envelopes in a spectral region where the noise dominates the transmittance spectrum. A flow chart of the algorithm of AEM is presented in Figure A1 in Appendix A. The execution of steps A2, A3, and A4 from the AEM algorithm allows analysis of the components of the total noise *N*(*λ*) and provides the preprocessed spectrum *T*_p_(*λ*) to be utilized henceforth.

The main problem of drawing *T*_+_(*λ*) and *T*_−_(*λ*) with *T*_c_(*λ*) << 0.3 is that the difference between the envelopes is considerable over a wide RWA and drops super-exponentially for *λ* < *λ*_g_, which hampers accurate determination of *λ*_t_(*λ*) in the RSA [35]. To resolve this problem, extensions in the RSA are computed, utilizing the iterative approach from [36], for each one of the two envelopes already prepared in the RWA, as mentioned in step A6 from the AEM algorithm. However, the extension to the higher envelope has a pair of upper boundary points, selected from the known higher envelope in the RWA, so that their wavelengths split in three the interval [*λ*_t_(*l*),*λ*_t_(*l* + 1)] where *λ*_t_(*l*) refers to the lowest or second lowest apparent maximum from the higher envelope in the RWA. The extension to the higher envelope also has a pair of lower boundary points selected from *T*_p_(*λ*), with *λ*_h_ > *λ*_p_ for the lower of these points, and the wavelength distance between this pair of points is the same as for the pair of the upper boundary points. Moreover, the extension to the lower envelope has a pair of upper boundary points with the same wavelengths as those for the pair of upper boundary points in the extension to the higher envelope, and the pair of lower boundary points is identical to these for the extension to the higher envelope. The upper point from the pair of lower boundary points represents the convergence point *T*_p_(*λ*_c_) for the extended envelopes.

Notably, the two optimized intersecting lines in the WD plot, described in step A9 from the AEM algorithm, correspond to two undamped oscillators influencing *T*_p_(*λ*) in its RWA.

The AEM utilizes the representation of *n*(*λ*) in the RSA by a second-order Cauchy equation:

(8) $$n\left(\lambda \right)=C_{1}+\frac{C_{2}}{\lambda^{2}}+\frac{C_{3}}{\lambda^{4}},$$

where *C*_1_, *C*_2_ and *C*_3_ are fitting parameters. In addition, the following new formula is derived for determination of *k*(*λ*) in RSA, by using the equations for *T*_p_(*λ*), *T*_+_(*λ*) and *T*_−_(*λ*) from [29], taking into account the substrate absorption:

(9) $$k\left(\lambda \right)=\frac{\lambda W_{1}\left(\lambda \right)}{8\pi }\left\{1-\sqrt{1-\frac{4W_{2}\left(\lambda \right)}{{\left[W_{1}\left(\lambda \right)\right]}^{2}}}\right\},$$

where

$$\begin{array}{l} W_{1}=\frac{{\left(\tau_{a,f}\tau_{f,s}\tau_{s,a}\right)}^{2}x_{s}}{{\rho_{a,f}}^{2}\left[{\rho_{f,s}}^{2}-{\left(\rho_{s,a}x_{s}\right)}^{2}\right]T_{i}},\;W_{2}=\frac{1-{\left(\rho_{f,s}\rho_{s,a}x_{s}\right)}^{2}}{{\rho_{a,f}}^{2}\left[{\rho_{f,s}}^{2}-{\left(\rho_{s,a}x_{s}\right)}^{2}\right]},\;T_{i}=\frac{2T_{+}T_{-}}{T_{+}+T_{-}},\\ \tau_{a,f}\tau_{f,s}\tau_{s,a}=\frac{8}{\left(n+1\right)}\frac{n}{\sqrt{{\left(n+n_{s}\right)}^{2}+{k_{s}}^{2}}}\sqrt{\frac{{n_{s}}^{2}+{k_{s}}^{2}}{{(n_{s}+1)}^{2}+{k_{s}}^{2}}},\;x_{s}=\frac{4\pi k_{s}}{\lambda },\\ \rho_{a,f}=\frac{n-1}{n+1},\;\rho_{f,s}=\sqrt{\frac{{\left(n-n_{s}\right)}^{2}+{k_{s}}^{2}}{{\left(n+n_{s}\right)}^{2}+{k_{s}}^{2}}},\;\rho_{s,a}=\sqrt{\frac{{(n_{s}\;-\;1)}^{2}+{k_{s}}^{2}}{{(n_{s}+1)}^{2}+{k_{s}}^{2}}}, \end{array}$$

as pointed out in step A11 from the algorithm of the AEM. Since the previously discussed extension of the envelopes *T*_+_(*λ*) and *T*_−_(*λ*) increases their accuracy, the extension should also increase the accuracy of *k* (*λ*) in the RSA from Equation (9), which implicitly includes *T*_i_(*λ*) and both envelopes.

Furthermore, step A13 in the algorithm employs the derivatives of log_10_(*αE*) vs. *E* and −d[log_10_(*αE*)]/d*E* vs. *E*, rather than the very dependencies described in [50]. This leads to the use of smoother curve with flat regions, which can provide more accurate energy intervals *IE* of dominance of electron transitions with a particular value of *q*. Thereafter, the tangent to the Tauc function, from the Tauc plot for this value of *q*, is drawn at *E* corresponding to the average of the considered derivatives over *IE*, which should result in more accurate calculation of the respective energy gap. This energy gap is determined as the photon energy at which this tangent, to the Tauc function, crosses the axis *E*.

### 3.2. Experimental Features

Three samples are employed in this research, each one of them consisting of rf magnetron sputtered thin a-Si film on 1 mm-thick Corning Glass Eagle XG substrate. Argon gas was used in the sputtering process, while the gas pressure was 0.1 Pa for sample A079, 0.7 Pa for sample A031 and 1.1 Pa for sample A072. Normal incidence transmittance spectra of these samples were measured by a Perkin-Elmer Lambda 1050 UV/visible/NIR double-beam spectrophotometer providing illuminated film area of 10 mm × 3 mm, as 1 nm wavelength step of *T*(*λ*) is utilized in the present study. In addition, the following detectors were used in the measurement of such spectra: PbS detector for *λ* = [1801,2500] nm, InGaAs detector for *λ* = [861,1800] nm, and PMT detector for *λ* = [200,860] nm; *λ* decreased during the measurement [54]. Unlike in [54], though, the measured transmittance spectrum *T*_s_(*λ*) and reflectance spectrum *R*_s_(*λ*) of a bare substrate are also used in this study.

### 3.3. Preprocessing of the Transmittance Spectra T(λ)

Outliers in *T*(*λ*) occur in many kinds of experimental datasets and can be removed, e.g., by the six sigma approach [58,59]. Regarding the general noise, our recent study demonstrated that superior denoising of UV-Vis-NIR spectra *T*(*λ*) of thin films is achieved by employing a method, abbreviated as SMEDM, based on complete ensemble empirical mode decomposition with adaptive noise (CEEMDAN) [60]. Also, spectrophotometers and spectroscopic ellipsometers usually contain monochromators with bandpass Δ*λ*(*λ*), resulting in the presence of bandpass noise [61]. Yet, such monochromators typically have a triangular bandpass function, which allows calculation of the bandpass noise by using a formula from [62].

**Figure 1:**
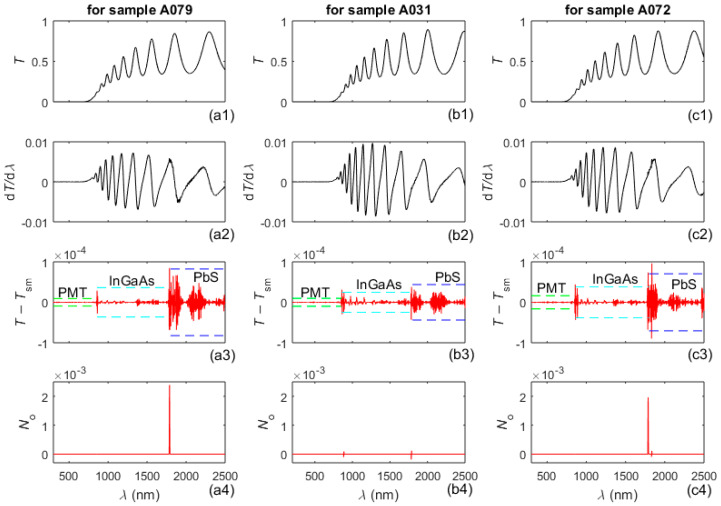
Plots related to the removal of outliers from *T*(*λ*) and calculation of the outlier noise *N*_o_(*λ*). The plots in the third row use *T*_sm_(*λ*) representing *T*(*λ*) smoothed by the Savitzky–Golay filter [63], as well as the six sigma limits of *T*(*λ*) − *T*_sm_(*λ*) over the three spectral ranges covered by the respective detectors.

**Figure 2:**
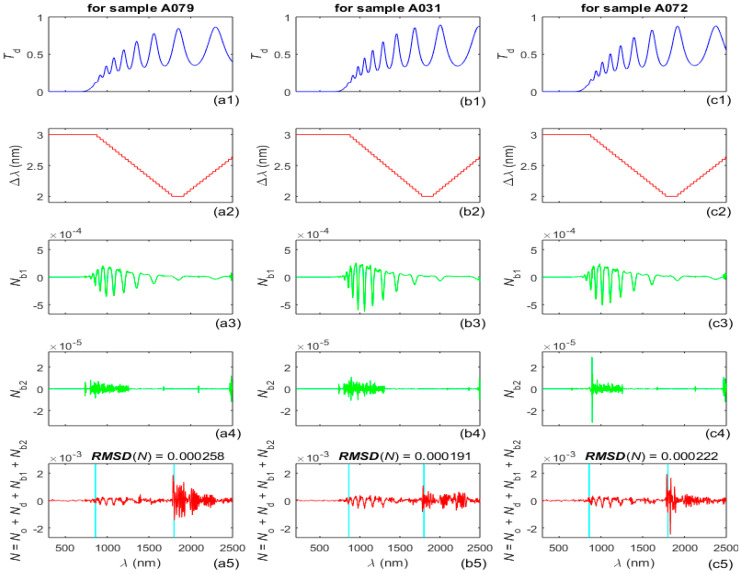
The spectra *T*_d_(*λ*) = *T*_o_(*λ*) − *N*_d_(*λ*) are in the first row of plots, the bandpass Δ*λ*(*λ*) used in the measurements of *T*(*λ*) is in the second row, the first and second components of the bandpass noise (calculated as in [62]) are in the third and fourth rows, and the total noise is in the last row.

**Figure 3:**
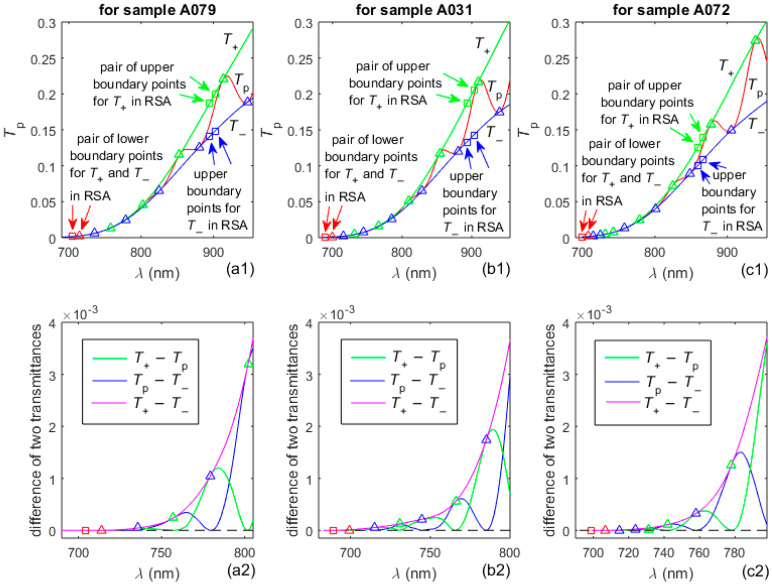
Plots regarding the extensions to both envelopes of *T*_p_(*λ*) into RSA; the black dashed line represents zero. The boundary points are exhibited by squares except for the convergence point *T*_p_(*λ*_c_) illustrated by red triangle. The tangency points *T*_+_(*λ*_t_) and *T*_−_(*λ*_t_), between *T*_p_(*λ*) and its extended envelopes, are represented by green and blue triangles, respectively.

**Figure 4:**
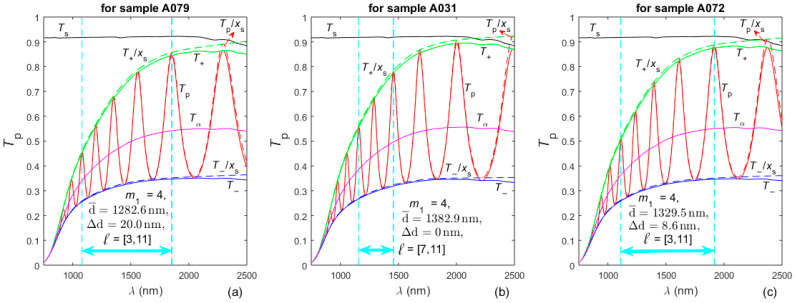
The spectrum *T*_p_/*x*_s_ and its envelopes *T*_+_/*x*_s_ and *T*_−_/*x*_s_ are displayed by dashed lines, while the spectrum *T*_p_(*λ*) and its envelopes *T*_+_ and *T*_−_ are drawn as solid lines. The OEM-computed optimized values of $\bar{d}$, ∆d, *m*_1_, and *l* = [*l*_1_,*l*_2_] are also included, and the wavelength interval corresponding to these *l* values is represented by double arrow.

**Figure 5:**
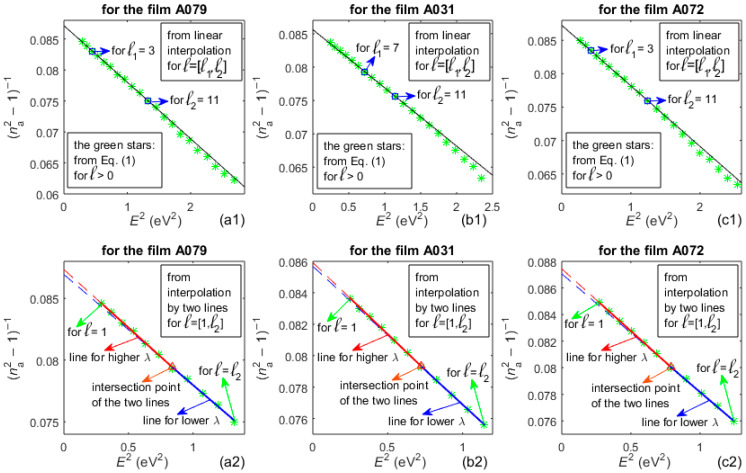
WD plots with green stars corresponding to *n*_a_(*λ*_t_). The first row from these plots refers to common fitting by one line of the dependence (*n*_a_^2^ − 1)^−1^ vs *E*^2^, only for *λ*_t_(*l* = [*l*_1_,*l*_2_]) obtained from OEM. The second row is for fitting by two intersecting lines of (*n*_a_^2^ − 1)^−1^ vs *E*^2^, only for *λ*_t_(*l* = [1,*l*_2_]).

**Figure 6:**
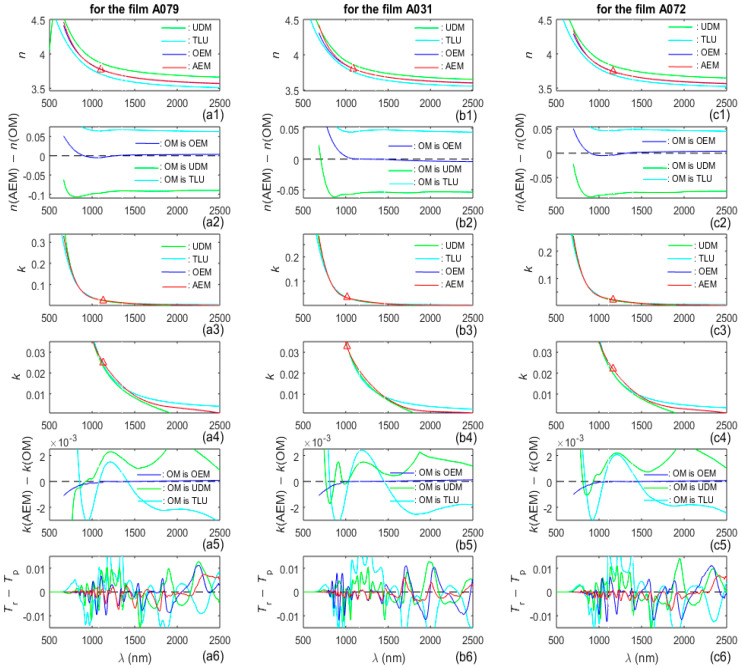
Drawings concerning *n*(*λ*) and *k*(*λ*) computed by TLU, UDM, OEM, and AEM. In the first row of graphs, the red triangles denote *n*(*λ*_n_), while the red triangles in the third and fourth row show *k*(*λ*_k_). The plots from the fourth row are magnified images, in RWA, of these from the third row. Each curve with a particular color from the sixth row corresponds to the method represented by a curve with the same color in the first row.

**Figure 7:**
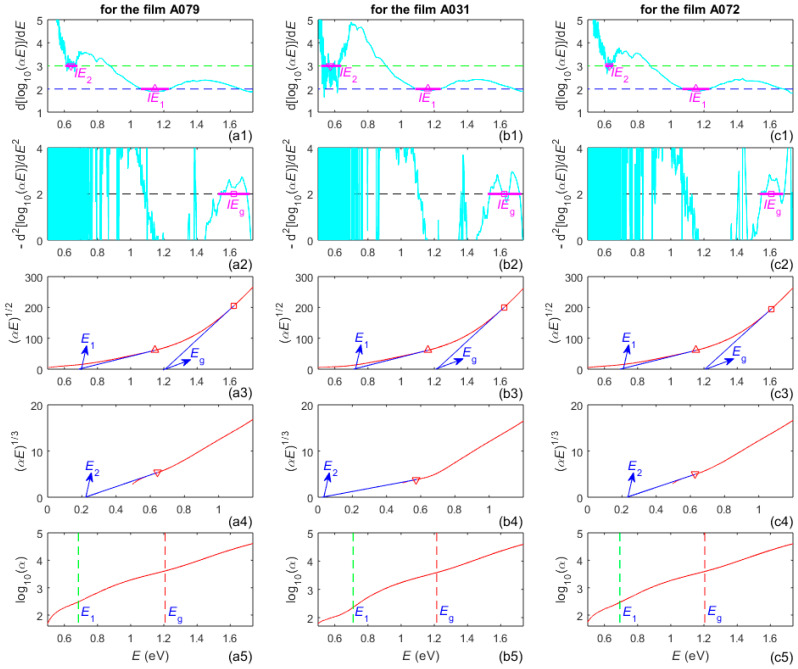
Graphs related to Tauc plots and Urbach plots using *k*(*λ*) obtained by AEM. The magenta segments from the panels in the first and second row represent *IE* for different *q* in Equation (3). Each magenta triangle or square in these panels corresponds to the photon energy *E* where the tangent is drawn to the respective Tauc function from the third or fourth row.

**Figure A2:**
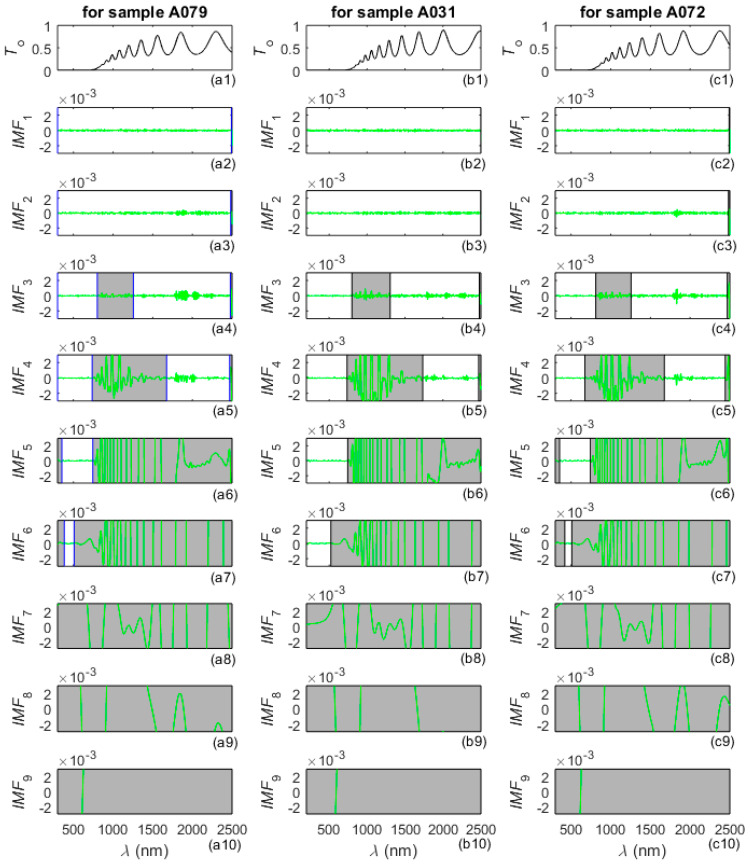
The spectra *T*_o_(*λ*) and *IMF*s from their decomposition by CEEMDAN. The areas with bumpy *IMF*_k_(*λ*) are shaded in gray and contain signal features, while those with flattish *IMF*_k_(*λ*) are in white and contain noise features, according to SMEDM [31].

**Figure A3:**
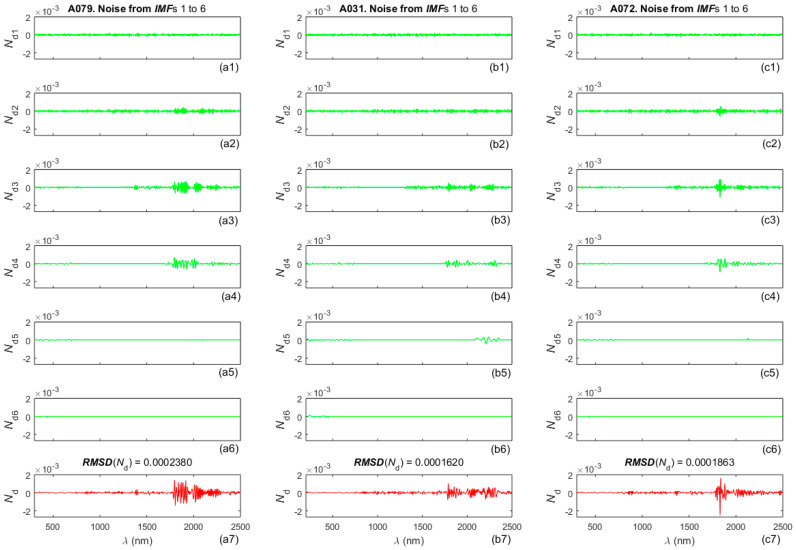
Components *N*_d1_(*λ*) to *N*_d6_(*λ*) of the general noise, extracted from their respective *IMF*s with at least one white area in Figure 3 (following SMEDM [31]), and the general noise *N*_d_(*λ*) drawn in red.

Graphs regarding characterization of the three studied a-Si films, by successive steps from the AEM algorithm in Figure A1, are presented in Figure 1, Figure 2, Figure 3, Figure 4, Figure 5, Figure 6, Figure 7, Figure A2 and Figure A3, where panels for A079 are in the first column, those for A031 are in the second, and those for A072 are in the third. With respect to the visualization of the results, in Figure 1, Figure 2, Figure 6, Figure A2 and Figure A3, the *x*-axis for each column is identical and labels are provided in the bottom row for clarity. The measured spectra *T*(*λ*), as well as results pertaining to the execution of step A2 from the AEM algorithm for determining the outlier noise *N*_o_(*λ*) and the spectra *T*_o_(*λ*) without outliers, are shown in Figure 1.

Following the results in [60], the decomposition of *T*_o_(*λ*) is performed using CEEMDAN with 3500 realizations of auxiliary white noise and a magnitude of 0.2 adjusting these realizations. The intrinsic mode functions (*IMF*s) obtained by such decomposition of *T*_o_(*λ*), and their spectral ranges with noise features, derived by SMEDM from [60], are illustrated in Figure A2 in Appendix A.

In Figure A3 in Appendix A3, we see the components of the general noise obtained from the *IMF*s with at least one area of noise features. The general noise *N*_d_(*λ*) is a sum of these components, as is also shown in Figure A3, where *RMSD*(*N*_d_) is the root mean square deviation of *N*_d_(*λ*) over the entire measured spectrum. Actually, the individual *IMF*s of *T*_o_(*λ*) differ for different runs of CEEMDAN; however, *N*_d_(*λ*) remains virtually constant, as demonstrated in [60].

Graphs illustrating the determination of the bandpass noise *N*_b_(*λ*) = *N*_b1_(*λ*) + *N*_b2_(*λ*) and the total noise *N*(*λ*), as described in step A4 from the AEM algorithm, are provided in Figure 2, where *RMSD*(*N*) is the root mean square deviation of *N*(*λ*) over the measured spectrum. Notably, the calculations of both the outlier noise *N*_o_(*λ*) and the bandpass noise *N*_b_(*λ*) do not employ adjustable parameters. The preprocessed spectrum *T*_p_(*λ*) = *T*(*λ*) − *N*(*λ*) is used extensively henceforth, for each one of the samples A079, A031 and A072.

### 3.4. Computation of Extended Envelopes and Non-Dispersion Parameters of the Spectra and the Films

The two envelopes in the RWA of *T*_p_(*λ*) are computed as in [36]. The new technique for extending these envelopes down to convergence point *T*_p_(*λ*_c_) << 0.03 from the RSA, mentioned in step A6 of the AEM algorithm, was described in Section 3.1. Graphs related to the preparation of the extensions to both envelopes into the RSA are shown in Figure 3.

OEM is performed as described in [42]. Graphs showing the extended envelopes *T*_+_(*λ*) and *T*_−_(*λ*) and results from the OEM are presented in Figure 4. As seen from Figure 4, *T*_s_(*λ*) decreases for *λ* > 2000 nm. This indicates that there is absorption in the substrate for these wavelengths, i.e., *k*_s_(*λ*) should be taken into account for accurate characterization of the three a-Si films, which was not done in [54]. With respect to this and a comment from Section 1, the curves *T*_+_(*λ*)/*x*_s_(*λ*) and *T*_−_(*λ*)/*x*_s_(*λ*) included in the double transformation of *T*_p_(*λ*) used in the OEM are not distorted for *λ* > 2000 nm; however, their corresponding envelopes *T*_+_(*λ*) and *T*_−_(*λ*) are distorted there. Notably, the results from the OEM indicate that the films A079 and A072 are non-uniform, since their ∆d > 0, although they were considered as being uniform in [54].

### 3.5. Results Regarding n(λ), k(λ), and f_voids_

According to the text from Section 1, the essence of step A8 from the AEM algorithm is illustrated by the graphs in the first row in Figure 5, while the graphs in the second row represent step A9 of this algorithm.

The dependencies *n*_WD_(*λ* = [*λ*_p_,max(*λ*)]) and *k*_WD_(*λ* = [*λ*_p_,max(*λ*)]) are calculated from these fitted lines in the WD plot and *T*_α_(*λ*), respectively, as described in steps A8 to A10 from the AEM algorithm. Next we compute *n*_SA_(*λ* ⊂ RSA) and *k*_SA_(*λ* ⊂ RSA), according to step A11, followed by determination of *n*(*λ* = [*λ*_p_,max(*λ*)]) and *k*(*λ* = [*λ*_p_,max(*λ*)]) as explained in step 12.

Furthermore, TLU as well as UDM parametrizations, including $\bar{d}$ and ∆d as unknowns, are performed by using *T*_p_(*λ*). In the TLU parametrizations, we assume the physically plausible boundary condition *ε*(*E* → ∞) = 1 for the dielectric function *ε*, as discussed in [51]. The employed UDM is based on the formalism from [52], and includes one excitonic term of interband transitions since the utilization of one-oscillator DMs has been considered successful for characterization, from *T*(*λ*), of other rf magnetron sputtered thin a-Si films [64,65]. In addition, high-energy excitations of valence electrons are excluded from the UDM, because such excitations occur at photon energies *E* > *E*_h_/4 + 3*E_g_*/4 which are higher than max(*E*) for our measured spectra. Instead, a pole, representing a high-energy Lorentz oscillator with vanishing broadening, is introduced in the UDM by adding a term *N*_p_/(*E*_p_^2^ − *E*^2^) to *ε*, as explained in the spectroscopic ellipsometry study [66]. The respectively computed TLU and UDM parameters are exhibited in Table 1.

Plots including *n*(*λ*) computed by the TLU, UDM, OEM, AEM and their mixed differences, as well as *k*(*λ*) computed by the TLU, UDM, OEM, AEM and their mixed differences, are included in Figure 6.

After completing a particular a-Si film characterization, the respective reconstructed spectrum *T*_r_(*λ*) can be computed, along with the corresponding *FOM*s from Equation (5), for different wavelength intervals. Such *FOM*s, for *λ* ⊂ [*λ*_p_,*λ*(*l*_2_)] representing RSA, *λ* ⊂ [*λ*(*l*_2_),*λ*(*l*_1_)] for intermediate absorption in the film (RIA), and *λ > λ*(*l*_1_) corresponding to the RWA, are included in Table 2.

The volume fractions *f*_void_ of voids in the a-Si films are calculated from Equation (7) by using *E*_0_, *E_d_*, and *n*_0_ corresponding to the dashed red lines from the plots in the second row of Figure 5, i.e., *n*(*λ*) computed by the AEM. The values of these parameters are shown in Table 3.

**Table 2:** Results regarding the accuracy of characterization of the a-Si films by several characterization methods. The data in the third column are derived from Equation (1).

Film	$\bar{d}$ (nm), Δd (nm); Source	RE ($\bar{d}$) (%); Source	Method	*FOM* [*λ*_l_,*λ*(*l*_2_)]	*FOM* [*λ*(*l*_2_),*λ*(*l*_1_)]	*FOM* [*λ > λ*(*l*_1_)]	*FOM* [*λ*_l_,max(*λ*)]
A079	1299, 0; EM from [54]	0.850; EM from [54]	OEM	2.80	4.23	4.56	4.20
			AEM	0.82	2.90	3.84	3.30
	1282.6, 20.0; AEM	0.0652; AEM	TLU	7.16	13.0	7.15	8.32
			UDM	3.19	6.37	6.00	5.60
A031	1359, 0; EM from [54]	0.662; EM from [54]	OEM	3.86	2.01	6.09	5.08
			AEM	1.09	1.59	2.33	1.96
	1382.9, 0; AEM	0.1028; AEM	TLU	8.81	15.4	10.2	10.9
			UDM	3.89	4.82	5.43	4.97
A072	1310, 0; EM from [54]	0.992; EM from [54]	OEM	3.89	3.51	5.64	4.99
			AEM	1.10	1.74	3.23	2.68
	1329.5, 8.6; AEM	0.0504; AEM	TLU	6.94	14.5	8.21	9.25
			UDM	3.53	6.25	6.21	5.72

**Table 3:** Computed data regarding the determination of the volume fractions *f*_void_ (%) of voids, based on the results for *n*(*λ*) derived by AEM.

Film	*E*_0_	*E* *_d_*	*n*_0_	*f*_void_ (%)
A079	3.04	34.9	3.53	6.68
A031	3.07	35.7	3.56	5.65
A072	3.06	35.0	3.53	6.75

Furthermore, Equation (6) is reworked for the cases when a film from a given material contains a second medium, e.g., consisting of volumes containing particular molecules or molecular complexes, instead of gaseous voids. The volume fraction *f*_sm_ of the second medium is derived similarly to Equation (7), whereby:

(10) $$f_{sm}\;≃\;\frac{\left({n_{z0}}^{2}+\;2{n_{0}}^{2}\right)\left({n_{y0}}^{2}-{n_{0}}^{2}\right)}{3{n_{0}}^{2}\left|{n_{y0}}^{2}-{n_{z0}}^{2}\right|}\times 100(\%),$$

where *n*_z0_ = *n*_z_(*E* → 0) is the static refractive index of the pure second medium. Equation (10) represents a generalization of Equation (7), and it is not used in this paper; however, it is available for future studies.

### 3.6. Results Related to k(λ) Computed by AEM

Several dependencies for determination of the dominant electron transitions in the three a-Si films, the energy intervals *IE* of dominance of these transitions, and their energy gaps, described in Section 1, are included in Figure 7.

The energy gap with largest value, amongst the three energy gaps from the third and fourth row in Figure 7, corresponds to the largest absorption coefficient *α*(*λ*). Therefore, it is the bandgap for electron transitions from the valence band to the conduction band, which is commonly designated as *E_g_*. Values of *q*, as well as their respective energy intervals *IE* and energy gaps, are calculated from Figure 7, and are presented in Table 4.

**Table 4:** Values of *q*, energy intervals *IE* of dominance of electron transitions with these values of *q*, and energy gaps *E*_2_, *E*_1_ and *E_g_* for these transitions.

Film	*q*	*IE*_2_ (eV)	*E*_2_ (eV)	*q*	*IE*_1_ (eV)	*E*_1_ (eV)	*q*	*IE*_g_ (eV)	*E_g_* (eV) from Figure 7	*E_g_* (eV) from [54]
A079	3	[0.612,0.672]	0.219	2	[1.07,1.22]	0.685	2	[1.53,1.71]	1.21	1.23
A031	3	[0.525,0.632]	0.021	2	[1.09,1.23]	0.716	2	[1.53,1.72]	1.21	1.24
A072	3	[0.611,0.647]	0.229	2	[1.08,1.23]	0.693	2	[1.54,1.67]	1.21	1.24

## 4. Discussion

A comparison between the noise components *N*_o_(*λ*), *N*_d_(*λ*), *N*_b_(*λ*), and the total noise *N*(*λ*), included in Figure 1, Figure 2, Figure A2 and Figure A3, indicate that the detectors are the main source of noise in *T*(*λ*) of the a-Si films, as the PbS detector introduces most noise, followed by the InGaAs detector. In addition, it is seen from the graphs in the third and fourth row of Figure 1 that spikes of outlier noise usually occur just below 1800 nm, i.e., after the replacement of the PbS detector by the InGaAs detector. The magnitude of the bandpass noise increases with decreasing distance between adjacent *λ*_t_, and this magnitude is significantly smaller than those for *N*_d_(*λ*) and *N*_o_(*λ*), as seen in Figure 1, Figure 2 and Figure A3. The plots from the last row of Figure 2 also show that the magnitude of the total noise is largest around 1800 nm, where the PbS detector is replaced by the InGaAs detector, whereby this magnitude does not exceed 2.5 × 10^−3^. Since the primary source of systematic measurement error in *T* (*λ*) relevant to this study is the very small stray light error, which is <1.5 × 10^−6^ [67], this error is disregarded in this paper.

In the preparation of the extended envelopes of *T*_p_(*λ*) convergence points *T*_p_(*λ*_c_) are achieved with the following values: 2.00 × 10^−3^ for A079, 4.47 × 10^−4^ for A031, and 1.38 × 10^−3^ for A072. These values do not exceed 2.00 × 10^−3^, indicating that in this study we attained the required drawing of envelopes with *T*_p_(*λ*_c_) << 0.03.

In EMs, $\bar{d}$ is computed first, followed by *n*(*λ*), and then *k*(*λ*), which means that accurate determination of $\bar{d}$ is needed for accurate characterization of thin films by envelope methods. With respect to this, the data from the third column of Table 2 indicate that the average value of RE($\bar{d}$) is 0.835% for the characterizations of the three a-Si films studied here, from [54], by the EM of Swanepoel for uniform film on transparent substrate [38]. From the same column in Table 2 we also see that the average value of RE($\bar{d}$) is 0.0728% for the characterizations of these films by the AEM proposed here. Therefore, the AEM leads on average to 11.5-times-smaller relative error in the computation of $\bar{d}$ for these a-Si films, with respect to the EM from [38]. In addition, the data from the second column in Table 2 show that using the AEM, compared to the EM from [38], results in the following relative differences in the computed $\bar{d}$: −1.26% for A079, 1.76% for A031 and 1.49% for A072. As a comparison, the thickness of the film A031 was measured by a Dektak surface profiler to be 1385 nm, and by SEM to be 1348 nm in [54], while this study provides that $\bar{d}$ = 1382.9 nm, as shown in Figure 4b.

As seen from the WD plots in the first row of Figure 5, the vast majority of the green stars in the RSA are positioned below the black line. This is due to the fact that the function (*n*^2^ − 1)^−1^ is inversely proportional to both *E_d_* and the intensity of the strong interband electron transitions, as seen in Equation (2). Similarly, the shallower slope of the blue lines in RIA compared to their respective red lines in the RWA, in the graphs from the second row in Figure 5, indicates a stronger intensity of the electron transitions via tail states than via dangling bond states. This shows that tail states have a higher concentration than dangling bond states in the studied a-Si films.

The fact that most green stars in the RSA in Figure 5 are located below the black line also indicates that *n*(*E*) should be larger in the RSA than the value used in the OEM. Indeed, *n*(AEM) > *n*(OEM) in the RSA, according to the respective lines from the first row of graphs in Figure 6. In addition, the graphs in the fourth row in Figure 6 illustrate that *k*(UDM) < 0 in the RWA and *k*(TLU) is significantly larger than *k*(AEM) ≈ 0 in the RWA. Since previous studies have shown that *k*(*λ*) ≈ 0 in the RWA of a-Si [42,68], the above results indicate that both the UDM and TLU provide inaccurate characterization of the a-Si films in the RWA.

In the text after Equation (5), it is commented that a smaller *FOM* calculated over some wavelength region corresponds to more accurate thin film characterization over this region. In connection with this, amongst the 48 *FOM* data, shown in the last four columns in Table 2, there are 12 for each of the four employed characterization methods; whereby these 12 *FOM* data refer to the three a-Si films and the four spectral regions covering all *λ* > *λ*_l_. A comparison in corresponding manner of the four sets of 12 *FOM* data, for these four methods, shows that utilizing the TLU leads to the largest values for all 12 *FOM* data, while the AEM provides the smallest values for all 12 *FOM* data. Moreover, the OEM gives 9 penultimate smallest *FOM* data and UDM—3 penultimate smallest *FOM* data. Furthermore, the ratio [*FOM*_av_(OEM) − *FOM*_av_(AEM)]/*FOM*_av_(OEM) ≈ 44.4%, where *FOM*_av_ is an average over the three films of the respective *FOM*s from the last column in Table 2, and represents a relative decrement in the error of characterization by the AEM compared to the OEM.

The results from the previous three paragraphs show that the AEM provides the most accurate characterization of the three a-Si films, in each one of the three studied regions from their spectra *T*_p_(*λ* > *λ*_p_), and the TLU provides the least accurate characterization of these films in these regions. Therefore, this justifies the use of *n*(*λ*) and *k*(*λ*), computed by the AEM, as inputs for the preparation of Table 3, Figure 7, and Table 4.

In respect to the above, the AEM results from the second column of Table 2 and the last row from Figure 2 demonstrate the following relationship for the three studied films: the thicker the film, the smaller its non-uniformity ∆d, and the smaller the noise *N*(*λ*) in its measured spectrum *T*(*λ*).

According to the data from Table 3, the studied a-Si films contain voids, with quite low volume fractions *f*_void_ = [5.65,6.75]%, unlike the conclusion from the less accurate characterization in [54] that these films do not contain voids. In addition, sensitivity analysis shows that *f*_void_ has similar sensitivities to errors in *n*_y0_ and *n*_0_. Moreover, the relative error in *f*_void_ increases almost linearly with errors in *n*_y0_ and *n*_0_, and this relative error does not exceed 14.2% when the absolute errors in both parameters remain below 0.01. With regard to this, results from [54] indicated that, in general, a decreasing volume fraction of voids *f*_void_ in magnetron sputtered a-Si films is associated with increases in both the static refractive index *n*_0_ and the extinction coefficient *k*(*λ*).

Notably, it has been concluded in [64] that the TLU with an Urbach tail formulated by an equivalent of Equation (4), as in [51], provides proper characterization of other magnetron sputtered a-Si films. Additionally, *FOM*[*λ*_l_,max(*λ*)] = [5.55,12.67], with *T*(*λ*) included in Equation (5) instead of *T*_p_(*λ*), was obtained in [64] for the same TLU applied to other a-Si films, which is commensurate with *FOM*[*λ*_l_,max(*λ*)] = [8.32,10.9] for this TLU, which is seen from the last column in Table 2. Since results from the present work already showed that this TLU leads to the least accurate characterizations, employing the Urbach tail expressed by Equation (4) over the entire subgap region is clearly inaccurate for the studied a-Si films. Taking into account the linearity of log_10_(*α*) vs. *E* < *E_g_* from Equation (4), this assumption is supported by the apparent absence of a straight line approximation of this dependence, for all *E* < *E_g_*, from the graphs in the last row of Figure 7.

Nevertheless, the linear parts of the curves in the graphs in the last row of Figure 7, for *E* just below *E_g_*, represent the Urbach tail region, where the electron transitions are via tail states. According to Equation (4), the Urbach energy *E*_U_ can be calculated from the slopes of these linear parts, yielding *E*_U_ = 288 meV for the film A079, *E*_U_ = 274 meV for A031, and *E*_U_ = 277 meV for A072. However, the non-linear parts of the above-mentioned curves, for *E* below the Urbach tail region, represent the dangling bond region, where the electron transitions occur mainly via dangling bond states.

**Figure 8:**
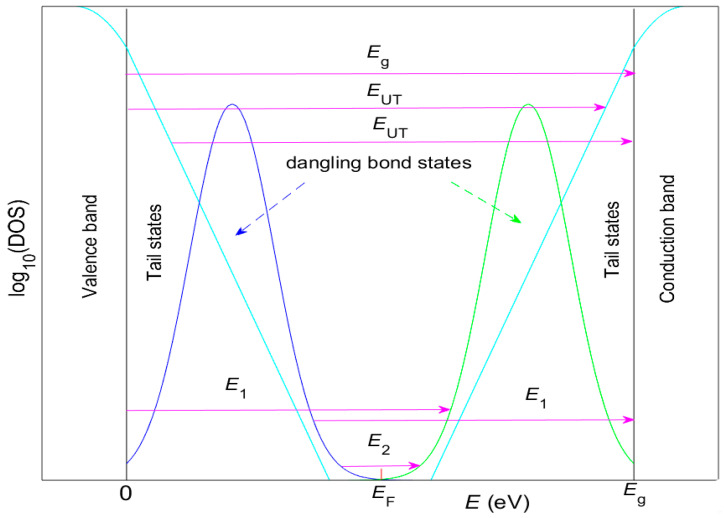
Sketch of DOS and the energy gaps *E*_1_, *E*_2_, and *E_g_* for the studied a-Si thin films.

Furthermore, the types of electron transitions featured in *T*(*λ*) are determined taking into account Equation (3), the graphs from the first two and last rows of Figure 7, and Table 4. Accordingly, these transitions are: indirect allowed transitions above the bandgap *E_g_* = 1.21 eV, indirect transitions via tail states with energy *E*_UT_ just below *E_g_*, indirect allowed transitions with energy gap *E*_1_ = [0.685,0.693] eV, and indirect forbidden transitions with energy gap *E*_2_ = [0.021,0.229] eV. In addition, the energy intervals *IE* of dominance of these electron transitions contain significantly higher energies compared to the energies of optical phonons in a-Si [68]. The above data can be explained by the drawing regarding the density of states (DOS) depicted in Figure 8.

The representation of the DOS in Figure 8 is consistent with the Mott–Davis model for amorphous materials [12], as well as with the involvement of electronic transitions via both tail states and dangling bonds. In this model, tail states are caused by structural disorder, because the bond angles and bond lengths vary slightly from the ideal crystalline values. This strains the network, pushing some states from the edges of the valence and conduction bands into the band gap. On the other hand, a dangling bond occurs in a semiconductor with predominantly tetrahedrally coordinated atoms, such as a-Si, when an atom has only three neighbors, leaving one electron unbonded. Dangling bonds create deep levels near the center of the band gap, where lower energy states correspond to dangling bonds occupied by single electrons and higher energy states represent dangling bonds occupied by two electrons [12]. Furthermore, the Fermi level *E*_F_ is fixed between the energies of these two types of dangling bond states, whereas the electronic states below *E*_F_ are mostly occupied, while those above *E*_F_ are mostly unoccupied prior to irradiation by a spectrophotometer’s light [14].

Importantly, electronic transitions via both tail states and dangling bond states have not been included in any DM utilized for spectroscopic characterization of a-Si from one measured UV-Vis-NIR spectrum, most likely because the concentration of dangling bond states in a-Si is significantly lower than that of tail states [68,69]. Furthermore, sequential parametrization can achieve a better accuracy of film characterization than simultaneous parametrization, provided it is feasible, due to improved convergence and reduced sparsity-induced instability [70]. Therefore, the successful analysis of transitions via dangling bonds in this study can be associated with the sequential computation of $\bar{d}$, ∆d, *n*(*λ*) and *k*(*λ*) by AEM, compared to the simultaneous computation of many model parameters by the dispersion model-based TLU and UDM.

In addition, another two magnetron sputtered a-Si films were also parametrized by the UDM, from a quasi-normal incidence reflectance spectrum *R*(*λ*) of the sample [71]. The lowest *FOM*s reported in [71] for these films were 5.18 and 5.46, respectively, which is significantly larger than the *FOM*s obtained in this paper by the AEM. Yet other magnetron sputtered a-Si films have been parametrized in [72] by reflectance spectroscopic ellipsometry (measuring the change in light’s polarization state) and the Cody–Lorentz dispersion model, thus not providing a *FOM* corresponding to Equation (5). Importantly, though, it has been assumed in both [71,72] that a single oscillator model describes the behavior of *T*(*λ* > *λ*_g_), which is shown to be insufficiently accurate by the present study.

The *FOM*s obtained by the AEM and reported in Table 2 are record lows, which indicates that the AEM can be used for improved design in relatively new practical applications of a-Si films. These include high-density memory devices based on spintronics [73,74], next-gen solar power (PV-EC systems for hydrogen) [75], advanced LiDAR for autonomous vehicles [76], silicon photonics for AI/data centers/telecoms (like 6G) [77], and improved battery anodes for EVs [78]. In addition, the AEM can be used to characterize thin films with different chemical compositions, e.g., graphene films similar to those from [79,80], as long as they provide an interference transmittance spectrum *T*(*λ*) with at least five extrema, in accordance with results from [41].

## 5. Conclusions

This paper reports the development of an advanced envelope method (AEM) for accurate characterization a single thin film on a thick substrate using its UV-Vis-NIR transmittance spectrum, *T*(*λ*). The AEM includes the following theoretical novelties: improved preprocessing of *T*(*λ*), drawing envelopes deeper into RSA, using two lines in the WD plot for the computation of *n*(*λ*) in the RWA, and employing both the Cauchy equation and a new formula for determination of *n*(*λ*) and *k*(*λ*) in the RSA.

It is demonstrated that the AEM provides most accurate characterization of three a-Si films, compared to the OEM, TLU and UDM; the relative decrement of the characterization error by the AEM is ≈44.4%, on average for these three films, compared to the OEM, which shows the second best results. In fact, the *FOM*s obtained from the AEM characterizations in this study are the lowest recorded compared with all previously published characterizations of magnetron sputtered thin a-Si films. This indicates that the most accurate characterizations of such films are the characterizations by the AEM reported here.

As a result of significantly decreased characterization errors in the AEM, especially for *k*(*λ*), four different types of electron transitions are identified in the three a-Si films. The respective energy gaps are calculated, and the DOS is interpreted to be consistent with the Mott–Davis model for amorphous semiconductors. Notably, analysis of electron transitions via dangling bonds and description of intricate DOSs have not been possible in the framework of the EM. Importantly, the AEM results reported here represent the first characterizations of a-Si films that account for electronic transitions via both the tail states and dangling bond states, using only spectroscopic ellipsometry or spectrophotometry in the UV-Vis-NIR region.

Since the studied a-Si films contain voids with the volume fraction *f*_void_ = [5.65, 6.75]%, they represent a nanostructured material with an internal nanoscale surface structure. In addition, *f*_void_ should be proportional to the concentration of dangling bonds in these films, as indicated in [81]. On the other hand, dangling bond states mostly act as non-radiative centers that trap charge and reduce mobility, unlike tail states [69,82]. These factors highlight the importance of the separate analysis of dangling bond states from tail states, as achieved in this research.

In future, the AEM can be used for characterization of thin films with different compositions, and it can also be expanded to include more oscillators in the RWA and RIA. Furthermore, the novel Equation (10) can be used for calculation of the volume fraction *f*_sm_ of the second medium (e.g., consisting of volumes containing particular molecules or molecular complexes), applicable not only for films, but for any mixture of two media.

## Figures and Tables

**Table 1:** Computed data from TLU and UDM parametrizations of the three a-Si films. The TLU parameters are designated by the same symbols as in [51], including A_0_ = 1, and the UDM parameters are denoted as their corresponding parameters from [52]. Each parametrization is considered complete once all its parameters stop changing to the fourth significant digit, within 300 steps of minimizing the figure of merit.

Film	DM Utilized in the Parametrization and its Computed Parameters						
A079	TLU with *ε*(*E* → ∞) = 1 as in [51]						
	*A* (eV)	*E*_0_ (eV)	*C* (eV)	*E_g_* (eV)	*E*_c_ (eV)	$\bar{d}$ (nm)	Δd (nm)
	101.7 ± 2.1%	3.442 ± 1.7%	1.628 ± 2.4%	1.064 ± 0.5%	1.585 ± 1.2%	1306 ± 0.6%	14.77 ± 3.2%
	UDM from [52] with one excitonic term and a pole						
	*N*_vc_	*E_g_* (eV)	*E*_h_ (eV)	*A*_1_	*E*_c_ (eV)	*B*_c_ (eV)	
	151.7 ± 1.4%	1.254 ± 0.2%	98.37 ± 3.2%	0.762 ± 2.1%	2.319 ± 3.0%	0.432 ± 3.1%	
	*N*_ut_	*E*_u_ (eV)	*N*_p_	*E*_p_ (eV)	$\bar{d}$ (nm)	Δd (nm)	
	50.20 ± 0.4%	0.2155 ± 0.3%	132.31 ± 2.3%	4.198 ± 1.1%	1250.8 ± 0.5%	26.75 ± 2.6%	
A031	TLU with *ε*(*E* → ∞) = 1 as in [51]						
	*A* (eV)	*E*_0_ (eV)	*C* (eV)	*E_g_* (eV)	*E*_c_ (eV)	$\bar{d}$ (nm)	Δd (nm)
	96.67 ± 1.8%	3.487 ± 2.0%	1.433 ± 2.9%	1.009 ± 0.3%	1.491 ± 1.4%	1400 ± 0.4%	0
	UDM from [52] with one excitonic term and a pole						
	*N*_vc_	*E_g_* (eV)	*E*_h_ (eV)	*A*_1_	*E*_c_ (eV)	*B*_c_ (eV)	
	201.2 ± 2.1%	1.324 ± 0.4%	13.85 ± 4.1%	0.555 ± 3.2%	2.725 ± 2.7%	0.285 ± 3.7%	
	*N*_ut_	*E*_u_ (eV)	*N*_p_	*E*_p_ (eV)	$\bar{d}$ (nm)	Δd (nm)	
	6.143 ± 0.9%	0.2210 ± 0.5%	256.33 ± 3.9%	8.888 ± 2.3%	1362.8 ± 0.4%	15.08 ± 5.1%	
A072	TLU with *ε*(*E* → ∞) = 1 as in [51]						
	*A* (eV)	*E*_0_ (eV)	*C* (eV)	*E_g_* (eV)	*E*_c_ (eV)	$\bar{d}$ (nm)	Δd (nm)
	100.0 ± 1.9%	3.457 ± 2.2%	1.564 ± 3.2%	1.046 ± 0.6%	1.547 ± 2.8%	1347 ± 0.9%	0
	UDM from [52] with one excitonic term and a pole						
	*N*_vc_	*E_g_* (eV)	*E*_h_ (eV)	*A*_1_	*E*_c_ (eV)	*B*_c_ (eV)	
	295.6 ± 2.4%	1.258 ± 0.6%	198.0 ± 3.8%	2.004 ± 2.9%	2.820 ± 3.3%	0.605 ± 3.2%	
	*N*_ut_	*E*_u_ (eV)	*N*_p_	*E*_p_ (eV)	$\bar{d}$ (nm)	Δd (nm)	
	103.8 ± 1.2%	0.2153 ± 0.7%	97.46 ± 4.2%	5.296 ± 1.7%	1300.5 ± 0.6%	19.39 ± 4.8%	

The data for TLU are in blue, and those for UDM are in green.

## Data Availability

The original contributions presented in this study are included in the article. Further inquiries can be sent to the corresponding author.

## Abbreviations

The following abbreviations are used in this manuscript:

CEEMDAN	complete ensemble empirical mode decomposition with adaptive noise
UV-Vis-NIR	ultraviolet–visible–near-infrared
EM	envelope method
RWA	region of weaker absorption in the film
RIA	region of intermediate absorption in the film
RSA	region of stronger absorption in the film
WD plot	Wemple–DiDomenico plot
DM	dispersion model
TLU	Tauc–Lorentz–Urbach dispersion model
UDM	universal dispersion model
OEM	optimizing envelope method
AEM	advanced envelope method
RMSD	root mean square deviation
FOM	figure of merit
RE	relative error
DOS	density of states
